# Development and evaluation of a novel real-time RT-PCR to detect foot-and-mouth disease viruses from the emerging A/ASIA/G-VII lineage

**DOI:** 10.1016/j.jviromet.2017.10.023

**Published:** 2018-02

**Authors:** Meruyert A. Saduakassova, Akhmetzhan A. Sultanov, Lespek B. Kutumbetov, Jemma Wadsworth, Britta A.Wood, Nick J. Knowles, Donald P. King, Katarzyna Bachanek-Bankowska

**Affiliations:** a Kazakh Scientific Research Veterinary Institute, 223 Raimbek Avenue, Almaty, 050016, Kazakhstan; b The Pirbright Institute, Ash Road, Pirbright, Woking, Surrey, GU24 0NF, United Kingdom

**Keywords:** FMDV, G-VII-lineage-specific assay

## Abstract

A new lineage of foot-and-mouth disease virus (FMDV), called A/ASIA/G-VII, emerged from the Indian subcontinent in 2015 and continues to spread in Western Asia. Currently, the distribution of viruses belonging to this lineage is defined using sequencing approaches, but other cheaper and faster diagnostic methods are urgently needed. Thus, this study describes the development and validation of a novel A/ASIA/G-VII lineage-specific real-time RT-PCR (rRT-PCR). Diagnostic sensitivity and specificity were evaluated using representative field specimens and isolates from the A/ASIA/G-VII lineage, as well as samples comprising other FMDV lineages that co-circulate in Asia (n = 54). This lineage-specific assay accurately detected all A/ASIA/G-VII samples tested (n = 29), and no detection was observed for samples belonging to other FMDV lineages (n = 25), namely A/ASIA/Sea-97, A/ASIA/Iran-05^SIS−10^, A/ASIA/Iran-05^FAR−11^, Asia1/ASIA/Sindh-08, O/CATHAY, O/ME-SA/PanAsia-2^ANT−10^, O/ME-SA/Ind-2001d, O/SEA/Mya-98. Additionally, the limit of detection was found to be at least equivalent to a pan-serotypic rRT-PCR assay. Therefore, these data indicate that this newly developed rRT-PCR assay can be applied to characterise field isolates in countries where the A/ASIA/G-VII lineage is endemic, as well as to monitor new incursions and outbreaks due to this lineage.

## 1 Type of research

Foot-and-mouth disease (FMD) is an infectious viral disease that affects cloven-hoofed animals. The causative agent of FMD is foot-and-mouth disease virus (FMDV), which is a positive-sense, single-stranded RNA virus classified in the genus *Aphthovirus* within the family *Picornaviridae.*

There are seven serotypes of FMDV (O, A, Asia 1, C, SAT 1, SAT 2 and SAT 3), each containing multiple genetic lineages. FMDV is not evenly distributed in endemic areas and its distribution can be divided into seven major ecological pools, with each pool containing different variants of the virus that are often restricted to a specific geographical location (Paton Sumption and Charleston, 2009). In Asia, three endemic pools have been identified (Pools 1–3) where serotypes A (lineages: ASIA/G-VII, ASIA/Sea-97, ASIA/Iran-05), O (lineages: ME-SA/Ind-2001, ME-SA/PanAsia-2, SEA/Mya-98, ME-SA/PanAsia, CATHAY) and Asia 1 (lineages: ASIA/Sindh-08, ASIA/G-VIII) co-circulate.

The A/ASIA/G-VII [also referred to as genotype 18 (Das et al., 2016)] lineage is classified within the A serotype, considered to be the most variable of the EurAsian serotypes. In 2015, viruses classified within this lineage were detected in Saudi Arabia, Armenia, Iran and Turkey, and continued to circulate in Saudi Arabia, Turkey and Iran in 2016 and 2017 (Bachanek-Bankowska et al., −submitted).

The ability to rapidly and accurately characterise FMDV lineages is necessary to understand the epidemiology of field outbreaks, as well as to aid the selection of appropriate vaccines. Lineage-specific real-time RT-PCR assays (rRT-PCR), targeting serotypic determinants encoded within variable VP1 (1D) sequences in the viral capsid, have been shown to be effective tools that can be used to detect and discriminate multiple FMDV lineages (Ahmed et al., 2012, Bachanek-Bankowska et al., 2016a, Jamal and Belsham, 2015, Knowles et al., 2016a, Reid et al., 2014). This study describes the development and evaluation of a new A/ASIA/G-VII lineage-specific rRT-PCR assay tailored to specifically detect viruses classified within this lineage.

## 2 Time required

The time required for this protocol is approximately 3.5 h, comprising of the following:

(i) 30 min for manual or automated nucleic acid extraction,
(ii) 30 min for master mix preparation, and
(iii) 2 h 30 min for amplification of target nucleic acid by one-step real-time RT-PCR.

## 3 Materials

### 3.1 Special equipment

Specialised equipment includes the following: Class II biological safety cabinet (e.g. Class II MSC, Walker); PCR instrument (e.g. 7500 Fast Real-Time PCR System, Applied Biosystems); extraction robot − optional (e.g. MagMax Sample Preparation System, ThermoFisher Scientific); and centrifuge (e.g. Heraeus Fresco 21, ThermoFisher Scientific).

### 3.2 Chemicals and reagents

Nucleic acid extraction kits: RNeasy MiniKit (Qiagen) for manual extraction or MagMAX- 96 Viral RNA Isolation Kit (ThermoFisher Scientific) for automated extraction; rRT-PCR kit: SuperScriptTM III Platinum^®^ One-Step qRT-PCR System (Invitrogen); A/ASIA/G-VII lineage-specific primers and dual-labelled probe (e.g. Sigma-Aldrich or equivalents) (Table 1).

**Table 1:** A/ASIA/G-VII specific primers and probes.

Oligo	aPosition	Nucleotide sequence (5′ → 3′)
G-VII_FP (sense)	465–482	TGCTCAACTCCCTGCCTC
G-VII_RP (antisense)	552–535	GAGTTCGGCACGCTTCAT
G-VII_P (sense)	506–524	FAM-CCACYACCATCCACGAGCTG-BHQ1

a Positions according to the VP1 coding sequence of the A/SAU/1/2015 sequence (KU127247).

## 4 Detailed procedure

### 4.1 Virus samples and preparation

A panel of FMDV clinical specimens (original suspensions and fluids; n = 29) and virus isolates (n = 25) was selected from the repository held at the World Reference Laboratory for FMD (WRLFMD) at the Pirbright Institute. FMD viruses in these samples had been previously characterised by VP1 sequencing (Knowles et al., 2016b) to belong to serotype A (lineages: ASIA/G-VII, ASIA/Sea-97, ASIA/Iran-05^SIS−10^, ASIA/Iran-05^FAR−11^), serotype O (lineages: ME-SA/Ind-2001, ME-SA/PanAsia-2^ANT−10^, SEA/Mya-98, CATHAY) and serotype Asia 1 (lineage: ASIA/Sindh-08) (Table 2). The panel comprised of: (i) ∼10% (w/v) original suspensions (OS) of vesicular epithelium (Ferris and Dawson, 1988) (n = 23), (ii) original fluid (OF) collected from unruptured vesicles (n = 5), and (iii) FMDV infected bovine thyroid cell culture supernatants (BTy) (n = 25) (Table 2).

**Table 2:** Diagnostic sensitivity and specificity of the G-VII- specific assay.

Sample name	Serotype/topotype	Lineage	Sample type	3D	G-VII
				Ct value	Ct value
IRN/8/2015	A/ASIA	G-VII	OS	16.47	14.57
IRN/12/2015	A/ASIA	G-VII	BTy1	11.33	6.73
IRN/14/2015	A/ASIA	G-VII	BTy1	12.27	9.11
IRN/18/2015	A/ASIA	G-VII	OS	16.21	15
IRN/21/2015	A/ASIA	G-VII	BTy1	12.43	9.47
IRN/1/2016	A/ASIA	G-VII	BTy1	9.83	9.87
IRN/8/2016	A/ASIA	G-VII	BTy1	11.03	7.68
IRN/11/2016	A/ASIA	G-VII	BTy1	11.4	7.11
IRN/11/2016	A/ASIA	G-VII	OS	20.99	19.1
IRN/12/2016	A/ASIA	G-VII	BTy1	11.51	8.19
IRN/20/2016	A/ASIA	G-VII	BTy1	11.86	7.78
IRN/23/2016	A/ASIA	G-VII	OS	22.11	19.89
IRN/39/2016	A/ASIA	G-VII	BTy1	16.87	15.28
IRN/4/2017	A/ASIA	G-VII	BTy1	17.24	16.3
SAU/3/2015	A/ASIA	G-VII	BTy1	14.81	12.51
SAU/4/2015	A/ASIA	G-VII	OS	20.19	16.58
SAU/8/2015	A/ASIA	G-VII	BTy1	14.21	8.79
SAU/9/2015	A/ASIA	G-VII	BTy1	12.37	8.92
SAU/14/2015	A/ASIA	G-VII	BTy1	19.44	16.11
SAU/15/2015	A/ASIA	G-VII	BTy1	24.76	21.47
SAU/16/2015	A/ASIA	G-VII	BTy1	14.18	10.81
SAU/17/2015	A/ASIA	G-VII	BTy1	15.74	10.78
SAU/21/2015	A/ASIA	G-VII	BTy1	18.66	15.14
SAU/15/2016	A/ASIA	G-VII	BTy1	11.39	7.83
SAU/15/2016	A/ASIA	G-VII	OS	36.32	27.96
SAU/19/2016	A/ASIA	G-VII	BTy1	18.88	17.29
SAU/20/2016	A/ASIA	G-VII	BTy1	18.98	16.9
SAU/22/2016	A/ASIA	G-VII	BTy1	16.88	14.67
SAU/40/2016	A/ASIA	G-VII	BTy1	12.89	10.94
AFG/5/2013	A/ASIA	Iran-05^FAR−11^	OS	21.68	No Ct
PAK/31/2015	A/ASIA	Iran-05^FAR−11^	OS	19.27	No Ct
TUR/31/2014	A/ASIA	Iran-05^SIS−10^	OS	25.84	No Ct
IRN/9/2015	A/ASIA	Iran-05^SIS−10^	OS	20.82	No Ct
MAY/15/2014	A/ASIA	Sea-97	OF	29.98	No Ct
VIT/9/2015	A/ASIA	Sea-97	BTY1	28.86	No Ct
VIT/10/2015	A/ASIA	Sea-97	BTY1	27.63	No Ct
AFG/6/2016	Asia 1/ASIA	Sindh-08	OS	24.83	No Ct
AFG/10/2016	Asia 1/ASIA	Sindh-08	OS	32.39	No Ct
AFG/21/2016	Asia 1/ASIA	Sindh-08	OS	24.5	No Ct
NEP/29/2015	O/ME-SA	Ind-2001d	OF	26.54	No Ct
MUR/1/2016	O/ME-SA	Ind-2001d	OS	27.13	No Ct
MUR/3/2016	O/ME-SA	Ind-2001d	OF	31.58	No Ct
NEP/5/2016	O/ME-SA	Ind-2001d	OF	34.49	No Ct
AFG/4/2016	O/ME-SA	PanAsia-2^ANT−10^	OS	28.65	No Ct
AFG/17/2016	O/ME-SA	PanAsia-2^ANT−10^	OS	27.25	No Ct
AFG/19/2016	O/ME-SA	PanAsia-2^ANT−10^	OS	25.68	No Ct
HKN/2/2016	O/CATHAY	–	OS	31.59	No Ct
HKN/3/2016	O/CATHAY	–	OS	30.21	No Ct
HKN/6/2016	O/CATHAY	–	OS	33.64	No Ct
HKN/7/2016	O/CATHAY	–	OS	36.18	No Ct
MYA/7/2016	O/SEA	Mya-98	OS	32.02	No Ct
MYA/9/2016	O/SEA	Mya-98	OS	27.98	No Ct
MYA/10/2016	O/SEA	Mya-98	OS	30.05	No Ct
TAI/33/2016	O/SEA	Mya-98	OF	30.71	No Ct

Diagnostic sensitivity tested with samples belonging to the A/ASIA/G-VII lineage (n = 29); diagnostic specificity tested with samples belonging to the A/ASIA/Sea-97 (n = 3), Asia 1/ASIA/Sindh-08 (n = 3), O/CATHAY (n = 4), O/ME-SA/PanAsia-2^ANT−10^ (n = 3), O/ME-SA/Ind-2001d (n = 4), O/SEA/Mya-98 (n = 4), A/ASIA/Iran-05^SIS−10^ (n = 2) and A/ASIA/Iran-05^FAR−11^ (n = 2) lineage.

BTy- bovine thyroid cell line; *OS- original suspension.

OF-original fluid; ME-SA- Middle East-South Asia; SEA- South East Asia.

### 4.2 RNA extraction

All samples were processed in a class II biological safety cabinet and the viral RNA was extracted using either the MagMAX 96 Viral RNA Isolation Kit (automated extraction) or RNeasy MiniKit (manual extraction). For automated extraction, 50 μl sample was mixed with 130 μl prepared lysis buffer supplied as a part of the MagMAX-96 Viral RNA Isolation Kit, while for manual extraction 460 μl sample was mixed with an equal volume of RLT lysis buffer supplied as a part of the RNeasy MiniKit. The viral RNA was stored at −70 °C until used in the experiments.

### 4.3 Primers and probe

The A/ASIA/G-VII-specific primers and probes were designed following identification of a unique lineage-specific target region in a nucleotide sequence alignment of the VP1 coding region of relevant sequences obtained from GenBank (http://www.ncbi.nlm.nih.gov/). All oligonucleotides were synthesised by Sigma-Aldrich (USA), and probes were labelled with BHQ-1 (Black Hole Quencher-1) and FAM at their respective 3′ and 5′ termini (Table 1).

### 4.4 Real-time RT-PCR protocol

The one-step rRT-PCR was carried out with 5 μl RNA in 25 μl total volume reaction mix containing 2 μl (10 μM) of each primer and 1.5 μl (5 μM) of the probe, 0.5 μl ROX (5 x concentration) 12.5 μl 2 × reaction buffer and 1 μl Superscript III/Platinum Taq enzyme mix (Life Technologies). Amplification conditions were identical to those used for pan-serotypic detection of FMDV (Reid et al., 2009, Shaw et al., 2007): 60 °C for 30 min, 95 °C for 10 min, followed by 50 cycles of 95 °C for 15 s and 60 °C for 1 h. Fluorescence was measured at the end of the 60 °C annealing/extension step. All reactions were performed in duplicate and amplified using the 7500 Fast Real-Time PCR System.

### 4.5 Protocol evaluation

The initial evaluation of the A/ASIA/G-VII lineage-specific assay was based on the comparison of the rRT-PCR results and the phylogenetic classification based on the VP1 coding sequence (WRLFMD, 2015a, WRLFMD, 2015b, WRLFMD, 2016a, WRLFMD, 2016b, WRLFMD, 2016c, WRLFMD, 2016d, WRLFMD, 2016e, WRLFMD, 2017a, WRLFMD, 2017b), followed by comparison with the results of the 3D pan-serotypic assay (Callahan et al., 2002). All samples were tested in duplicate by the lineage-specific and pan-serotypic rRT-PCRs. The amplification efficiency of the rRT-PCRs was assessed on the basis of a 10-fold dilution series (from 10^−2^ to 10^−7^) of a representative sample belonging to the A/ASIA/G-VII lineage (IRN/12/2015) in a diluent consisting of total nucleic acid extracted from a 10% epithelial suspension prepared from tongue tissue of a healthy cattle. These reactions were performed in duplicate. The rRT-PCR efficiency was calculated by using the following formula: Eff% = 10^−1/slope^ × 100.

## 5 Results

### 5.1 Diagnostic sensitivity and specificity

Diagnostic specificity and sensitivity of the A/ASIA/G-VII lineage-specific rRT-PCR assay were evaluated with a panel of 54 RNA samples using the pan-serotypic rRT-PCR assay as the reference test (Callahan et al., 2002) (Table 2). All samples, previously characterised by phylogenetic analyses of the VP1-coding sequence as belonging to the A/ASIA/G-VII lineage (n = 29), produced a positive rRT-PCR result (a Ct value) for both the lineage-specific and the 3D pan-serotypic rRT-PCR. The Ct values for the A/ASIA/G-VII lineage-specific assay were on average lower by 3.09 (t_1,27_ = 10.1, p = 0.000) than those obtained with the 3D pan-serotypic assay (Table 2). In addition, 25 RNA samples representing FMDV lineages other than the A/ASIA/G-VII and circulating in the same geographic area (pools 1 and 3) were tested to evaluate the assay for the diagnostic specificity. All these samples tested positive by pan-serotypic assay (Callahan et al., 2002), but produced no Ct result with the A/ASIA/G-VII-specific assay in each case (Table 2).

### 5.2 Limit of detection

**Fig. 1:**
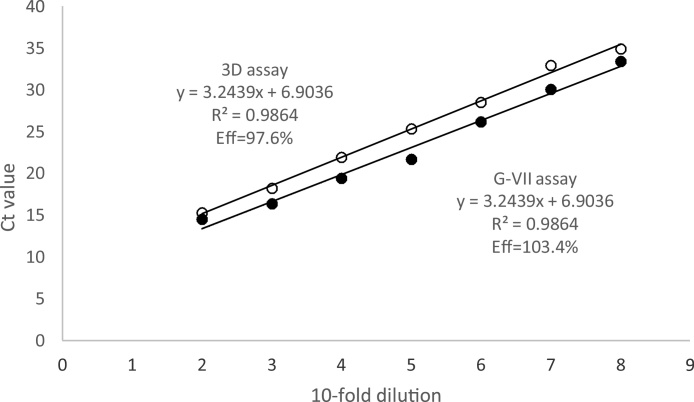
Comparative limit of detection of the A/ASIA/G-VII- and 3D pan-serotypic rRT-PCR assays. RNA dilution series of IRN/12/2015 sample was tested in duplicates with the 3D pan-serotypic assay (○) and the G-VII lineage-specific assay (●).

The relative limit of detection for the A/ASIA/G-VII specific rRT-PCR was assessed by examining ten-fold RNA dilution series of the IRN/12/2015 sample, in duplicate and in parallel with the 3D pan-serotypic assay. The efficiency of the A/ASIA/G-VII-specific assay was calculated to be 103.4%, higher than that obtained for the 3D pan-serotypic assay (97.6%) (Fig. 1).

## 6 Discussion

After the emergence of the A/ASIA/G-VII lineage into the Middle East in 2015 (Bachanek-Bankowska et al., 2016b), the lineage spread rapidly into multiple countries (Saudi Arabia, Armenia, Iran and Turkey) and, despite control efforts, it continues to circulate in this geographical area (Bachanek-Bankowska et al., 2015 −submitted). Recently, a similar epidemiological event has been reported for the O/ME-SA/Ind-2001d lineage, which also has emerged from the Indian sub-continent and continues to expand its circulation in Asia and the Middle East (Knowles et al., 2016a, Saravanan et al., 2015, Vu et al., 2017). Therefore, there is a need to rapidly and accurately identify samples that contain the A/ASIA/G-VII lineage and to distinguish them from other co-circulating FMDV lineages.

Given that the VP1 coding region varies according to a FMDV serotype and lineage, phylogenetic comparisons of the VP1 coding sequences are routinely used to characterise field viruses for epidemiological purposes (Abdul-Hamid et al., 2011, Knowles et al., 2016b, Subramaniam et al., 2015). This knowledge was previously exploited to generate rRT-PCR assays that can detect contemporary FMDV lineages circulating in the Middle East and West Eurasia: O/ME-SA/PanAsia-2, A/ASIA/Iran-05, Asia 1/ASIA/Group1, 2, 6 and 7 (Jamal and Belsham, 2015, Reid et al., 2014), East Africa: A/AFRICA/G-I, O/EA-2, O/EA-4, SAT1/I and SAT2/IV (Bachanek-Bankowska et al., 2016a), as well as for detection of individual lineages: SAT2/VII and O/ME-SA/Ind-2001 (Ahmed et al., 2012, Knowles et al., 2016a). Expanding the range of tests that are available, this study describes the design and validation of a new A/ASIA/G-VII-specific assay.

This A/ASIA/G-VII-specific rRT-PCR assay was shown to have high diagnostic sensitivity and correctly identified all viruses as belonging to the A/ASIA/G-VII lineage with Ct values lower than those of the pan-serotypic test detecting the 3D coding region. In addition, the genetic material from other FMDV lineages investigated in this study was not amplified, indicating high diagnostic specificity of this assay. Therefore, the application of the A/ASIA/G-VII lineage-specific assay provides a reliable method to rapidly characterise the A/ASIA/G-VII lineage. The G-VII lineage-specific rRT-PCR assay targets the most variable region of the genome making it possible that, with time, the target region will change, altering the assay’s specificity and/or sensitivity. Thus, it is important to monitor the performance of the lineage-specific assays over time, and it is recommended that all lineage-specific assays are used in combination with a pan-serotypic diagnostic approach to ensure that the presence of FMDV is correctly identified. The composition and the thermal profile of the G-VII-specific and the 3D pan-serotypic assays (Shaw et al., 2007) were designed to the same specifications, such that both can be performed at the same time and run in parallel using the same PCR cycling instrument.

The application of lineage-specific assays provides a relatively accessible method for FMDV lineage detection to laboratories which do not possess sequencing capacities. The development of this A/ASIA/G-VII-specific assay expands the portfolio (or molecular toolbox) of the published lineage-specific rRT-PCR assays enabling national laboratories in endemic settings to rapidly and accurately characterise this emerging lineage. This can promote rapid and accurate implementing control strategies.

## 7 Essential literature references

(i) Callahan et al. (2002)
(ii) Shaw et al. (2007)
(iii) Jamal and Belsham (2015)

## 8 Quick procedure

• For automated RNA extraction, add 50 μl sample (tissue culture supernatant, original fluid or original suspension) to 130 μl MagMAX-96 Viral RNA Isolation Kit Lysis Buffer (ThermoFisher Scientific). Use the MagMAX-96 Viral RNA Isolation Kit (ThermoFisher Scientific) with the MagMAX Sample Preparation System (ThermoFisher Scientific) and follow the manufacturer’s protocol.
• Alternatively, for manual extraction, add 460 μl sample (tissue culture supernatant, original fluid or original suspension) to 460 μl RNeasy Kit RLT Buffer (Qiagen) and follow manufacturer’s protocol.
• Use the SuperScript™ III Platinum^®^ One-Step qRT-PCR System (Invitrogen) to prepare the reaction mix with the custom made G-VII-specific primers and probe (Table 1) according to the protocol described in Section 4 (Detailed procedure).
• Perform rRT-PCR programme as described, time required: maximum 2 h 30 min
